# Use of PET-CT in diagnostic workup of periprosthetic infection of hip and knee joints: significance in detecting additional infectious focus

**DOI:** 10.1007/s00264-021-05218-8

**Published:** 2021-10-07

**Authors:** E. Roschke, T. Kluge, F. Stallkamp, A. Roth, D. Zajonz, K. T. Hoffmann, O. Sabri, R. Kluge, M. Ghanem

**Affiliations:** 1 Klinik Und Poliklinik Für Orthopädie, Unfallchirurgie Und Plastische Chirurgie, Universitätsklinikum Leipzig, Liebigstr. 20, 04103 Leipzig, Germany; 2 Klinik Und Poliklinik Für Nuklearmedizin, Universitätsklinikum Leipzig, Leipzig, Germany; 3 Klinik Und Poliklinik Für Diagnostische Und Interventionelle Radiologie, Institut Für Neuroradiologie, Leipzig, Germany; 4 Klinik Für Orthopädie, Unfallchirurgie Und Wiederherstellungschirurgie, Zeißigwaldkliniken Bethanien Chemnitz, Chemnitz, Germany

**Keywords:** Periprosthetic infection, Hip and knee arthroplasty, PET-CT

## Abstract

**Introduction:**

The diagnosis and management of periprosthetic knee and hip infections as well as the identification and management of possible additional infectious foci is of great importance for successful therapy. This study analyses the importance of ^18^F deoxyglucose PET-CT (PET-CT) in the identification of additional infectious focus and subsequent impact on management of periprosthetic infection (PPI).

**Material and methods:**

A retrospective analysis of the clinical data and findings in the period from January 2008 to December 2018 was carried out. One hundred and four patients with in-hospital treatment due to PPI of a hip or knee joint were identified and included in this study. All patients underwent a standardized clinical examination and further surgical and antibiotic therapy. The reevaluation of performed PET-CTs was specifically carried out with regard to the local PPI or detection of secondary foci.

**Results:**

PET-CT successfully verified the PPI in 84.2% of the patients. A total of 78 possible additional foci were detected in PET-CT in 56 (53.8%) of the examined patients. Predilection sites for possible secondary foci were joints (42.3%), pulmonary (15.4%), ear-nose-throat (15.4%), spine (11.5%), and the musculocutaneous tissues (11.5%). Fifty-four positive PET-CT findings were confirmed clinically with need of additional adequate treatment.

**Conclusion:**

PET-CT is a valuable diagnostic tool to confirm periprosthetic joint infection. At the same time, the whole-body PET/CT may detect additional foci of infection with impact on subsequent treatment strategy. PET was of special value in detecting infections at distant locations far from the primary infected joint in significant number. These distant infection locations can be potential cause of a re-infection. This clearly reflects the need of their diagnosis.

## Introduction

The endoprosthetic knee and hip replacement provides patients with pain relief, improved quality of life, and mobility. The frequency of periprosthetic infections (PPIs) is 1% for total hip replacement THR and 2% for total knee replacement TKR [1, 2]. If revision surgery is necessary, the infection rates increase with THR up to 3% and with TKR up to 5% [1, 2]. Despite an all-over low primary infection rate, with increasing number of primary hip and knee arthroplasty of more than 430,000 per year in Germany [3], PPI pose a challenge to both, patients and orthopaedic surgeons. PPI remains a major complication [4]. Treatment strategies mainly depend on the time of occurrence of PPI, soft tissue condition, the type of pathogen, and the clinical experience.

Infection must be correctly diagnosed and classified for appropriate therapy. Early acute infection is to be distinguished from late chronic infection [1].

The structured procedure for the diagnosis of PPI focuses on symptoms and signs of clinical examination, blood tests, imaging, microbiological, and histopathological evaluation of the joint puncture and intra-operative findings. The identification of pathogens in samples taken intra-operatively from the affected joint is of particular importance with high sensitivity and specificity [5–7].

According to literature reports, early-onset PPI are usually swiftly controlled by surgical revision and antibiotic treatment [1, 2, 6, 7]. Treatment of late infections is still challenging and most commonly arise due to haematogenous spread [8]. Still, thorough clinical examination in both cases may not be sufficient in detecting extra-articular sources of infection although the identification and treatment of additional infectious foci is of particular importance concerning eradication of periprosthetic infection.

As a whole-body examination, ^18^F deoxyglucose PET-CT (PET-CT) enables the precise localization of the increased glucose metabolism in infectious processes [9–11]. However, it is still not part of the standard diagnostic procedures. According to literature reports, there is no standardized battery of investigations; especially concerning the use PET in diagnosis and planning for management.

The aim of this retrospective study was to analyze the importance of PET-CTs in the diagnosis of PPI of the hip and knee joints, especially in identifying additional infectious foci and subsequent implications on therapeutic management.

## Material and methods

This study was approved by the Ethics Committee of the Medical Faculty of the University of ***Leipzig*** (vote-number 079/18-e). According to the ***Saxony*** University Act, data collection is allowed. Therefore, no informed consent is required.

**Fig. 1:**
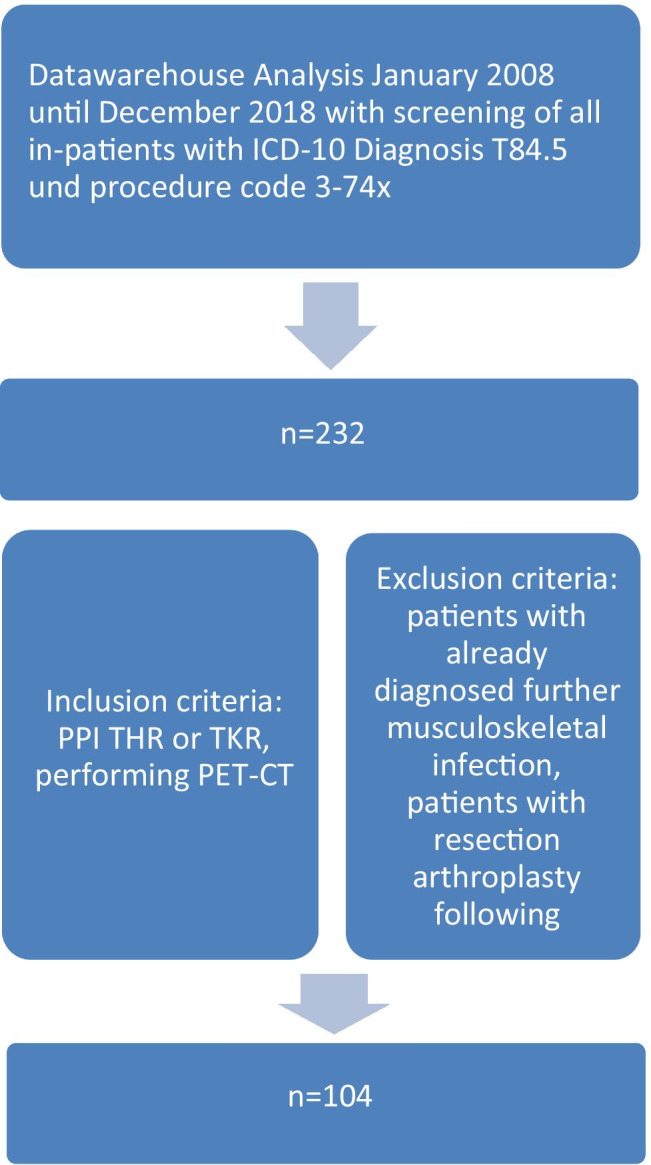
Patient enrollment

We retrospectively analyzed the clinical data and findings in the period from January 2008 to December 2018. In accordance with the criteria published by Parvizi in 2013 [6], the diagnosis of the PPI of the hip or knee joint was based on clinical symptoms and signs, microbiological and histopathological findings of joint puncture, and intra-operative samples. Two hundred and thirty-two patients were identified and were treated in our department due to PPI of the hip or knee joints during this time following the department guideline for PPI. We excluded those patients with already diagnosed further infection focus of the musculoskeletal organs as well as patients who already undergone explantation of THR or TKR (Fig. 1), so that 104 patients were ultimately included in the study.

Upon admission to hospital, all patients underwent a standardized clinical examination (including anamnesis of dental status and exclusion of urogenital or dermal infection) and further surgical and antibiotic therapy. Primarily, in 56 cases PET-CT was conducted to search for additional infectious foci, in 33 cases PET-CT primary was performed to estimate the extent of local PPI, and in 15 cases it was indicated to evaluate both issues. Ninety-one patients received a whole-body scan, 13 a partial scan including pelvis and lower extremities.

The assessment of a prosthesis infection in PET-CT was made visually by looking closely at the ^18^F-FDG uptake pattern. In THR, ^18^F-FDG uptake at the middle portion of the femoral component or in the periprosthetic soft tissues, except near to the greater trochanter can indicate an infection. ^18^F-FDG uptake in the lateral and medial sides of the acetabular cup, around the neck of the prosthesis, in the proximal or very distal portion of the component can be non-specific. In TKR, a ^18^F-FDG uptake at the bone-prosthesis interface or in the periprosthetic soft tissues is suspicious for prosthesis infection, while a synovial ^18^F-FDG uptake is rather non-specific [12]. For this analysis, all available PET-CT scans from enrolled patients were re-evaluated from two independent specialists in nuclear medicine focusing only on detection of additional infectious focus.

The PET-CTs were carried out in accordance with the guidelines of the German Society of Nuclear Medicine and were acquired on a Siemens Biograph 6 (Siemens AG Healthcare Sector, Erlangen, Germany). The PET-CT data were evaluated with the Hybrid Viewer (Version 3.0.5), Hermes Medical Solutions. The data collection was based on the treatment data and examination results stored in IS-H-SAP (Siemens AG Healthcare Sector, Erlangen, Germany).

The data acquisition and analysis was carried out using Microsoft Word Excel (Microsoft ©) and GraphPad Prism 8.4.1 (Graphpad Software, Inc).

## Results

The patient cohort examined had an average age of 70.3 ± 10.5 years, with female patients (53.8%) and periprosthetic THR infections (53.8%) being marginally more represented (Table 1).

**Table 1:** Cohort data

Cohort data			
	Total	TKR	THR
Sample size	104 (100%)	48 (46.2%)	56 (53.8%)
Age in years	70.3 ± 10.5	70.1 ± 9.3	70.4 ± 11.7
Men	48 (46.2%)	22 (45.8%)	26 (54.2%)
Women	56 (53.8%)	26 (46.4%)	30 (53.6%)
Pre-operative PET-CTs - With evidence of local infection	57 (54.8%)	30 (52.6%)	27 (47.4%)
	48 (46.2%)	26 (54.2%)	22 (45.8%)
Patients with additional infectious focus	56 (53.8%)	26 (46.4%)	30 (53.6%)

Out of 104 patients, a total of 48 periprosthetic TKR infections could be included, whereby 54.2% (26) female and 45.8% (22) male patients were affected. A similar gender distribution was found in 56 periprosthetic THR infections (53.6% (30) female and 46.4% (26) male patients).

When performed prior to surgery, PET-CT successfully verified the PPI in 84.2% of the patients. There were discrete differences depending on the joint affected (total knee replacement TKR vs total hip replacement THR—87.1% vs. 81.5%). When PET-CT was performed postoperatively, signs of PPI were still detected in 65.2% of patients. These differences were significant in Wilcoxon Mann–Whitney test (*p* < 0.05).

**Fig. 2:**
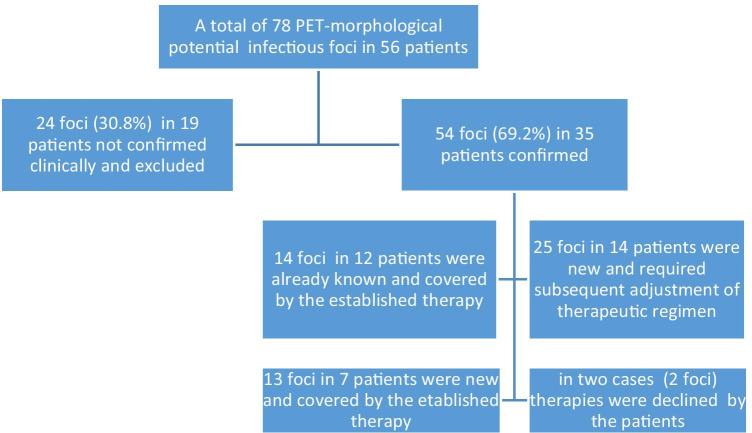
Overview of the newly detected additional infectious foci and their management

A total of 78 possible additional infectious foci were detected in PET-CT in 56 (53.8%) of the examined patients (Fig. 2).

Predilection sites for possible additional foci were joints (33; 42.3%), pulmonary (12; 15.4%), ear-nose-throat (ENT) and dental (11 ENT, 1 dental; 15.4%), spine (9; 11.5%), musculocutaneous tissues (9; 11.5%), and the gastrointestinal tract (3; 3.9%) (Table 2).

**Table 2:** A total of 78 PET-morphological evidence of additional focus of infection detected in 56 patients

Joints	Pulmonary infiltrates	Ear, nose and throat (ENT) and dental	Gastrointestinal	Musculocutaneous tissue	Spine
33 (42.3%)	12 (15.4%)	12 (15.4%) 11 ENT, 1 dental	3 (3.9%)	9 (11.5%)	9 (11.5%)

Concerning joints, elevated ^18^F-FDG uptake was seen in 11 distant artificial joints, ten native joints of the lower extremity, and 12 native joints of the upper extremity, where the shoulder joint (8/33, 24.2%) was the most common.

**Fig. 3:**
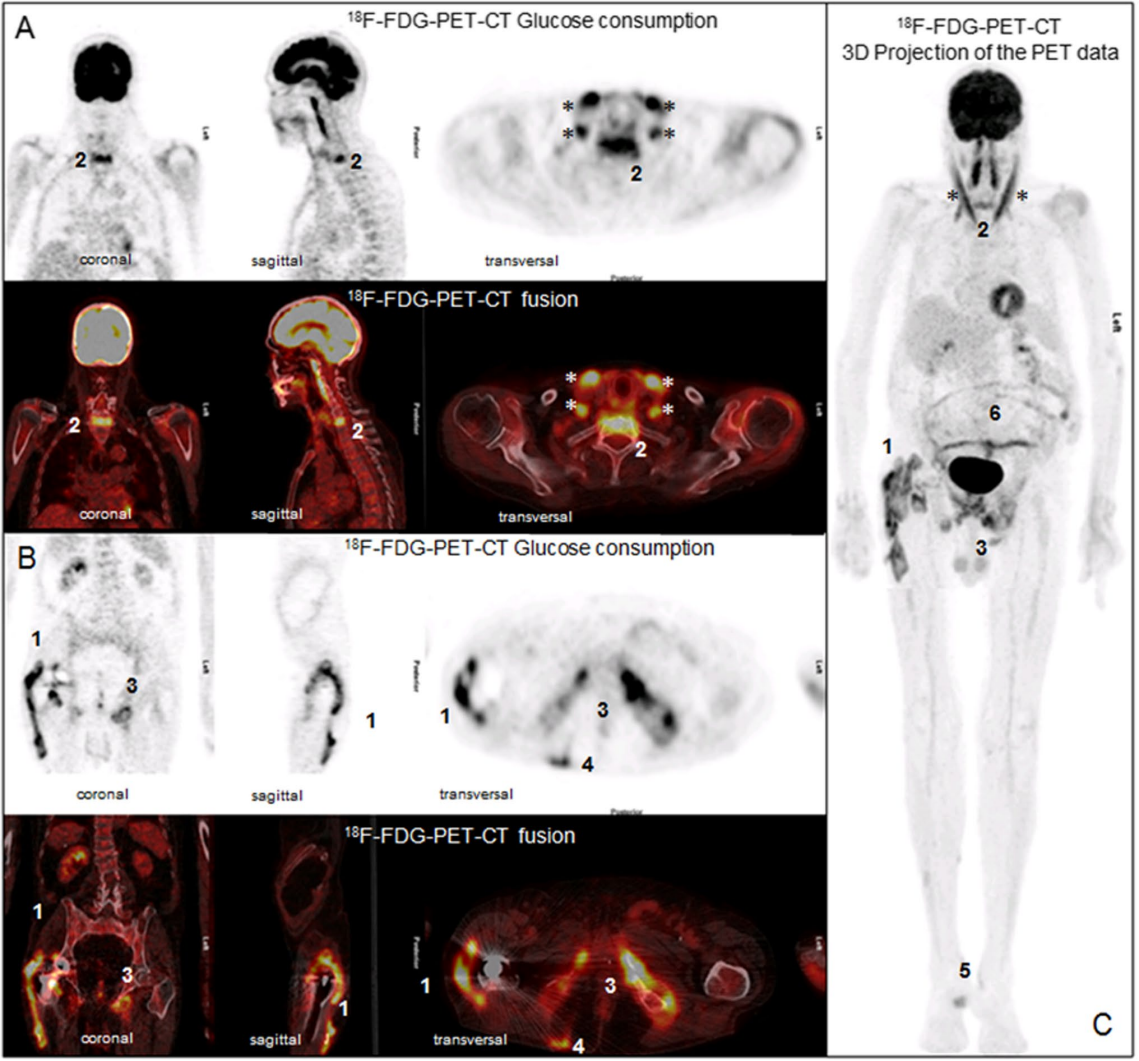
Representative PET-CT images of **A** head and thorax, **B** abdomen and pelvis in coronal, sagittal, and transversal orientation. Panel **C** shows a 3 D projection of the ^18^F-FDG-PET data. (1) Patient with infection in the right total hip arthroplasty with intensive ^18^F-FDG uptake in the periprosthetic soft tissues next the acetabulum, on both sides of the neck of the prosthesis and along the whole lateral part of the femoral component with also extensive infiltration into the surrounding periprostetic soft tissues. Additional ^18^F-FDG-PET-positive findings: (2) spondylodiscitis in C7/ Th1; (3) fracture of the left os oschii, left and right os pubis; infectious focus in the surrounding soft tissues; (4) fistula along the right M. gluteus maximus to the skin surface; (5) soft tissue infection at right calcaneaus; (6) enteritis of the small intestine. (*) Physiologic ^18^F-FDG uptake in neck muscules

In case of musculocutaneous infectious focus, 9 patients had a distant location from the affected joint. Eight spondylodiscitis and one paravertebral abscess were detected (Fig. 3).

Fourteen (17.9%) additional infectious foci in 12 patients were already known due to clinical examination or prior diagnostic/ imaging and were further confirmed by PET-CT. In all other 64 cases (82.1%), the potential additional infectious foci were clinically silent. As they could not be detected upon clinical examination, further diagnostic workup such as x-ray radiographs or laboratory investigations was necessary. After that, 24 (30.8%) positive PET-CT findings were not confirmed clinically. They could be excluded and no further treatment was needed (Fig. 2). Concerning the remaining foci, a sufficient treatment was carried out. Finally, 54 (69.2%) of all 78 foci needed further treatment.

In detail, in 12 patients 14 infectious foci were already known and treated by the already established therapy. The same antibiotic treatment that was applied for the primary joint infection was suitable in all these cases. In seven patients with 13 foci, the therapy was covered from antibiotic treatment of the periprosthetic joint infection and no further surgery was necessary. Two patients with two foci declined further treatment, and in 14 patients with 25 secondary foci a significant additional therapy was carried out.

## Discussion

The structured procedure for the diagnosis of PPI focuses on symptoms and signs of clinical examination, blood tests, imaging, microbiological, and histopathological evaluation of the joint puncture and intra-operative findings [1, 6, 7]. PET/CT is currently not part of the standard diagnostic algorithm. This method, however, might add relevant additional diagnostic accuracy with regard to different facettes of the disease:

1. To diagnose/rule out a floride infection and to evaluate the extension of the infectious process
2. To identify additional infectious foci

According to our review of literature, no prior study has dealt with the use of PET-CT for detecting additional infectious foci in periprosthetic infection and subsequent implication on management.

### PET-CT for detection of PPI

Several literature reports showed a sensitivity of 80–100% and specificity of 90–100% of PET/CT in the diagnosis of PPI of hip and knee joints [12, 13]. Using special uptake patterns, PET-CT can distinguish between septic and aseptic loosening [12–14]. The pooled sensitivity and specificity in current meta-analyses were 86% and 93% for THR [15] and 70% and 84% for TKR [16]. Our study could reassure this fact: typical signs of THR or TKR infection were seen in 85% of pre-operatively performed PET/CT examinations. Thus, PET-CT confirmed to be a valuable diagnostic tool in detecting PPIs in THR and TKR. Added value (increased accuracy) of PET/CT to conventional tests (including radiography, erythrocyte sedimentation rate [ESR]/C-reactive protein [CRP] testing, and joint aspiration culture and white blood cell count) in diagnosing PPI was confirmed by Kwee et al. with special benefit in cases without evident clinical symptoms, such as fistula, or previous surgery [17].

Concerning timing of conducting PET-CT, Zimmerli et al. indicated that the detection of PPI may be compromised in the early post-operative period. If PPI is suspected after surgery, PET-CT should not be carried out earlier than six to eight weeks post-operatively [2]. In our study, typical signs of THR or TKR infection were seen in 65.2% of cases when PET-CT was performed post-operatively. This underlines this recommendation [2].

### PET-CT for detecting additional infectious foci

One of the most common causes of PPI is hematogenous spread from other septic locations [17, 18]. Therefore, identification and restoration of infectious foci is crucial for successful treatment of PPI and avoidance of re-infection. The standardized diagnostic and therapeutic algorithm focuses on infections involving the skin and the urinary tract and on dental foci. Additional infectious foci often remain undetected in routine diagnostic workup. As a whole-body examination PET/CT may detect such foci and this is an accepted approach in patients with fever of unknown origin or with sepsis [19, 20]. However, to the best of our knowledge, no studies did specifically deal with the impact of a diagnostic workup using PET-CT in artificial joint replacement to detect additional places with infections elsewhere than the affected artificial joint and subsequent impact on treatment strategy.

Therefore, we focused in this study on the role of PET-CT in identifying additional infectious foci and subsequent implications on management strategy. A total of as much as 78 possible additional infectious foci were detected in PET-CT in 56 (53.8%) of the examined patients (Fig. 2 and Table 1). Fifty-four foci were confirmed and the therapeutic management was modified accordingly.

Only 14 (17.9%) foci in 12 patients were already known due to clinical examination or prior diagnostic/ imaging. In the remaining 40 foci in 23 patients, the additional infectious foci were clinically silent and could not be detected prior to performing PET-CT. Thus, in more than 20% of the patients, the conventional routine diagnostic algorithm failed to identify all infectious foci and PET-CT added therapeutically relevant information.

According to our literature research, we found no comparable studies that dealt with or evaluated the significance of PET-CT in the detection of additional infection foci as part of diagnostic investigations in the management of PPI after THR and TKR. Possible extra-articular sources of infection are frequently pulmonary, in the ENT region, dental, gastrointestinal as well as soft tissues and other joints [8, 21–23]. This was confirmed by the result of the PET-CT examination in our study. As a whole-body examination, the PET-CT is a single examination tool that enables the detection of multiple possible sources of infection.

To detect such additional infectious, foci complementary diagnostic investigations such as, e.g., abdominal ultrasound, chest x-ray, and endoscopic examinations must be carried out. Such a battery of investigation during hospital stay is time-consuming, expensive, and requires strict organization and suitable infrastructure.

In accordance with literature reports [6], we noticed in this study that the results of PET-CT examination have significant impact on planning of surgical interventions and make a targeted therapeutic approach possible. Untreated or unknown additional infectious foci can be one explanation for recurrence of periprosthetic infections after one- or two-stage revision in 9–15% of the cases [24–26]. Further studies are needed to confirm this hypothesis.

The limitation of this study lies in its retrospective design and the inclusion of a relatively low number of patients and the lack of a control group. Furthermore, PET-CTs were performed in many cases prior to surgery, yet in other cases after surgical intervention on the infected joint. Although all CTs were examined again by one independent investigator for the detection of additional distant foci, this may have caused a selection bias, as PET-CTs may have been performed in severe and complicated cases of PPI. As some of the PET-CTs focused on diagnosing the PPI, in some case the treating surgeon was not aware of extra-articular infections, which were detected subsequently at the retrospective evaluation of the scans. Furthermore, this study did not differentiated between acute and chronic PPI.

Yet, according to our results, we recommend considering PET-CTs as a significant supplement to standard diagnostic procedures in cases of periprosthetic joint infection after THR or TKR, especially when recurrent PPI are encountered. In order to avoid positive enhancement following joint surgery, we recommend performing PET-CT prior to surgical intervention, whenever possible. The search of additional infectious foci should be explicitely mentioned from the surgeon as a second important question to be answered by the PET/CT examination and the reporting nuclear physician should carefully check the complete whole-body scan for such findings.

## Conclusion

PET-CT is a valuable diagnostic tool with high diagnostic accuracy in the detection of periprosthetic joint infection after THR or TKR. Simultaneously, our results show for the first time the significance of PET-CT in detection of additional infectious foci and the subsequent adjustment of treatment strategy. PET-CT is of particular importance in detecting infection of further joints, the spine or in soft tissues, which remained undetected with the standard clinical examination protocol.

## Abbreviations

^18^F-FDG-PET-CT: 18 Fluorodeoxyglucose -positron- emission-computer tomography
PET-CT: ^18^ Flurorodeoxyglucose-positron- emission-computer tomography
^18^F-FDG: 18 Fluorodeoxyglucose
CRP: C-reactive protein
DD: Differential diagnosis
ENT: Ear-nose-throat
ESR: Erythrocyte sedimentation rate
PPI: PPI
SUV: Standardized uptake value
THR: Total hip replacement
TKR: Total knee replacement
ZESBO: Zentrum zur Erforschung der Stütz- und Bewegungsorgane
